# A Treatment Dilemma: Bentall vs Transcatheter Paravalvular Leak Closure with Aortic Aneurysm Repair in a High Surgical Risk Patient

**DOI:** 10.7759/cureus.43765

**Published:** 2023-08-19

**Authors:** Saad Ur Rahman, Krishna Prasad, Muhammad Shariq Akram, Naveed Adoni, Sanjay Mehta

**Affiliations:** 1 Internal Medicine, Carle Illinois College of Medicine, Urbana, USA; 2 Internal Medicine, Carle Health, Urbana, USA; 3 Cardiology, Carle Foundation Hospital, Urbana, USA; 4 Internal Medicine, King Edward Medical University (KEMU), Lahore, PAK; 5 Interventional Cardiology, Carle Foundation Hospital, Urbana, USA

**Keywords:** amplatzer plug device, thoracic aortic aneurysm repair, bentall procedure, paravalvular leaks, endovascular aneurysm repair

## Abstract

This case presentation involves an 80-year-old male with a history of surgically repaired patent ductus arteriosus and surgical aortic valve replacement due to infective endocarditis, who presented with progressive heart failure symptoms and was found to have a severe aortic paravalvular leak (PVL) and ascending thoracic aortic aneurysm. Due to complex surgical anatomy and multiple chronic comorbidities, he was considered a poor candidate for traditional valve replacement surgery including the Bentall procedure. Instead, a multidisciplinary team opted for transcatheter paravalvular leak closure (TPLC) with an Amplatzer plug followed by planned endovascular aortic aneurysm repair. The patient showed significant improvement in symptoms and reduction in aneurysm size post-procedure leading to avoidance of the open-heart surgery. This case highlights the effectiveness of the percutaneous approach in high-risk surgical patients with PVL and complex anatomical considerations.

## Introduction

Paravalvular leak (PVL) is a significant complication after cardiac valve replacement procedures [1]. The two viable treatment approaches available for PVL are transcatheter paravalvular leak closure (TPLC) with Amplatzer plug insertion and traditional valve replacement involving redo sternotomy, which is a high-risk procedure [2]. While the Bentall procedure combines aortic root and valve replacement in a single open-heart surgery, its risk profile becomes notably elevated when performed on patients who are not well-suited for surgical intervention [3]. 

We present an intriguing case of a patient with complex surgical anatomy, diagnosed later with a severe aortic PVL and an ascending thoracic aortic aneurysm. The patient successfully underwent Amplatzer plug insertion leading to significant improvement in the ascending aortic aneurysm. This minimally invasive transcatheter approach not only helped in avoiding open-heart surgery, but highlighted the importance of diagnostic workup, the critical clinical decision-making process, and the treatment plan for this complex patient scenario.

## Case presentation

An 80-year-old male with a past medical history of patent ductus arteriosus requiring repair at age seven and 18, endocarditis at age 68 complicated with aortic regurgitation leading to bioprosthetic valve placement, paroxysmal atrial fibrillation, and heart failure with mildly reduced ejection fraction (45-50%) presented to the cardiology clinic for progressively worsening heart failure symptoms. He was transferred to the hospital for further evaluation. His initial laboratory workup was normal.

**Figure 1:**
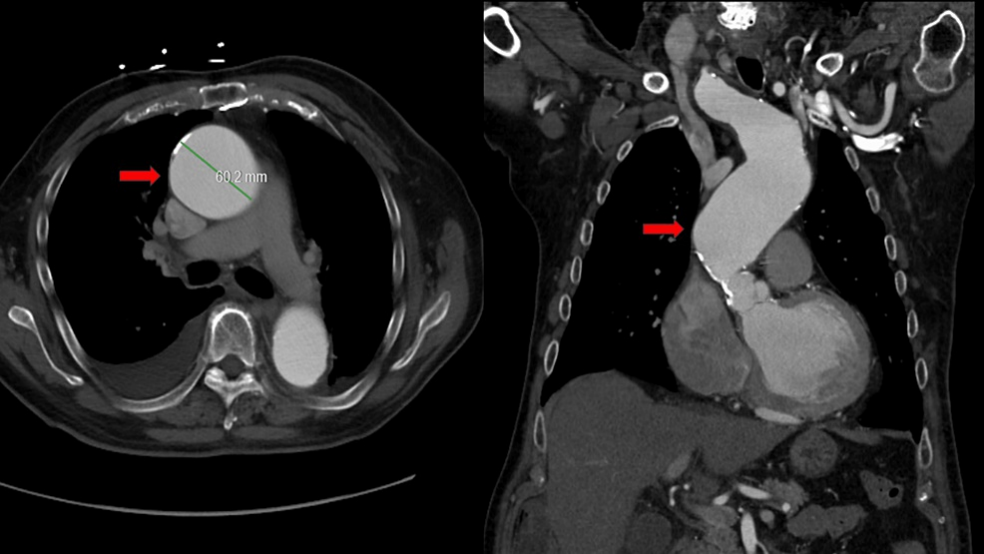
CTA chest showing thoracic aortic aneurysm measuring 6 cm and not involving aortic arch or arch vessels (red arrow pointing toward aneurysm) CTA, computed tomography angiography

The patient initially underwent a computed tomography angiography (CTA) chest that came back remarkable for a thoracic aortic aneurysm of 6 cm, not involving the aortic arch or arch vessels (Figure 1).

**Figure 2:**
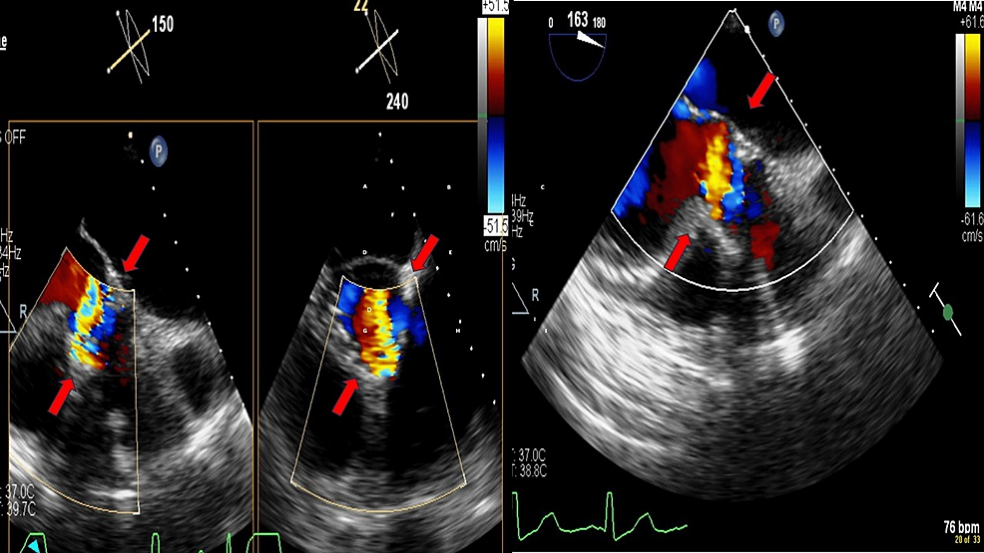
TEE demonstrating severe PVL, anterior to the bioprosthetic aortic valve ​(red arrows pointing) TEE, transesophageal echocardiogram; PVL, paravalvular leak

To further evaluate the symptoms, a transthoracic echocardiogram (TTE) was performed that showed moderate-to-severe aortic PVL. A transesophageal echocardiogram (TEE) was performed to further evaluate the function of his aortic valve, which showed a severe PVL at the 6 o'clock position in the area of the right coronary sinus, anterior to the previous bioprosthetic aortic valve. There was a very mild central valvular insufficiency (Figure 2).

Due to complex anatomy, multiple comorbidities, and three prior sternotomies, the patient was deemed a poor surgical candidate for the Bentall procedure. The case was discussed in a multidisciplinary approach involving cardiothoracic surgery and the interventional cardiology team. Given that the bioprosthetic valve was functioning well, it was decided to treat PVL with an Amplatzer plug following which the patient will be evaluated by cardiothoracic surgery for elective endovascular repair of thoracic aortic aneurysm. This hybrid approach will not only help avoid a high-risk open-heart surgery but will provide a viable less invasive treatment approach as well. 

**Figure 3:**
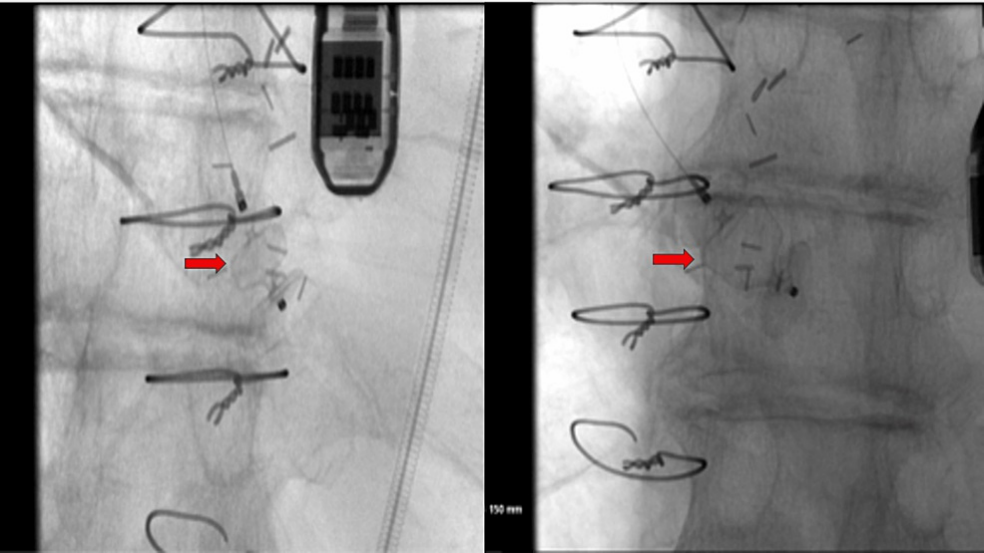
Left heart catheterization confirming accurate Amplatzer plug insertion (red arrows)

**Figure 4:**
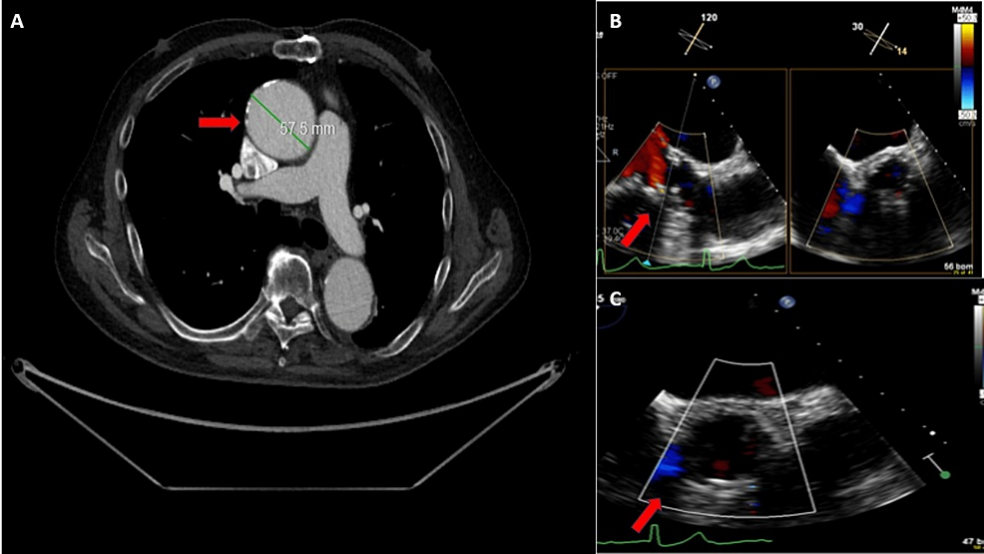
A. Post-op CTA showing slight improvement in AAA (red arrow). B. Post-op TEE showing significant improvement in the PVL (red arrow). C. Three-month follow-up TEE showing trace PVL (red arrow). CTA, computed tomography angiography; TEE, transesophageal echocardiogram; PVL, paravalvular leak; AAA, abdominal aortic aneurysm

The patient was educated about the procedure, and informed consent was taken. PVL was successfully treated with a 10×7 mm Amplatzer vascular plug with trivial PVL post-procedure (Figure 3). The patient also demonstrated significant improvement in his symptoms on three months follow-up post-procedure and a slight decrease in the diameter of the aneurysm, now measuring 5.7 cm (Figure 4). Thereafter, the patient was referred to cardiothoracic surgery for consideration of the repair of a stable ascending aortic aneurysm.

Given the considerable enhancement in the patient's clinical condition and the stability or even slight improvement in the thoracic aortic aneurysm, the cardiothoracic surgery team opted to continue with a conservative management strategy. The patient has been regularly attending follow-up appointments with the cardiothoracic surgery team for the subsequent 12 months, during which the thoracic aortic aneurysm has remained stable and asymptomatic.

## Discussion

PVL occurs in approximately 2-10% of patients with bioprosthetic aortic valves [4]. These leaks are commonly mild and often linked to surgical factors within the first year following valve replacement. Severe PVL, which may manifest as heart failure symptoms, infective endocarditis, and hemolytic anemia, affects about 1-5% of PVL cases and requires intervention [5]. Diagnosis and planning for management involve various imaging techniques such as TTE, TEE, and cardiac CT [6]. A thorough assessment of PVL should encompass the precise site, size, and orientation of the defect in relation to the sewing ring and the prosthetic valve occluders [7]. 

Limited data exist on the conservative management of PVL, with interventions often required due to high event rates. While medical therapy, including standard heart failure management, can alleviate symptoms, it does not prevent the progression of heart failure [6]. In between two viable options to treat PVLs, surgical reoperation is associated with high mortality and recurrence rates [4]. Also, long-term survival after surgical correction remains poor. The other treatment option is the percutaneous approach, which includes utilizing oblong closure devices. It has proven to result in improved leak severity and New York Heart Association (NYHA) classification, indicating its effectiveness [4]. Based on the current guidelines issued by the American Heart Association and American College of Cardiology, the percutaneous closure of PVLs is considered a reasonable approach (class IIA recommendation) for patients who exhibit significant regurgitation and are experiencing NYHA class III-IV symptoms, particularly for those who are at high risk for surgery due to complex underlying anatomy and other coexisting medical conditions [6]. 

Mortality predictors for PVL intervention include baseline creatinine level and NYHA functional class at presentation. Additionally, the extent of residual leak during follow-up evaluation correlates with major adverse cardiac events (MACE) and mortality [4]. 

In our case, the patient had two options. The first one would include the Bentall procedure which would address both aortic aneurysms and PVL. The second one would be to treat PVL with a percutaneous procedure and address a stable thoracic aortic aneurysm later. Our case highlights the importance of a multidisciplinary approach to complex clinical decision-making and a hybrid approach to the management of structural heart disease. In patients who have the pathology of ascending aorta and the valve, percutaneous management can be considered for the valve with an abbreviated surgical procedure for the aorta.

## Conclusions

PVL is an infrequent complication after valve replacement. TPVL closure with Amplatzer plug insertion has emerged as a secure, efficient, and minimally invasive substitute for surgical re-treatment. For patients unsuitable for surgery, serious consideration should be given to employing the Amplatzer plug. Successful management hinges on a collaborative, multidisciplinary approach coupled with informed shared decision-making.
